# Occupational Physical Activity in Young Adults and Stroke: Was It Due to My Job?

**DOI:** 10.7759/cureus.3217

**Published:** 2018-08-28

**Authors:** Tarig H Balla Abdalla, Ian H Rutkofsky, Javeria N Syeda, Zahid Saghir, Adnan S Muhammad

**Affiliations:** 1 Department of Research, California Institute of Behavioral Neurosciences & Psychology, Fairfield, CA, USA; 2 Medicine, International American University College of Medicine, Washington, USA; 3 Department of Research, California Institute of Behavioral Neurosciences & Psychology, Fairfield, USA

**Keywords:** stroke recovery, stroke rehabilitation, physical activity, stroke, stroke physical activity, working young adult stroke, young rehabilitation, stroke in young adults, working post-stroke, young adult stroke

## Abstract

The association of physical activity and stroke among working young adults and vice versa has increasingly empathized in recent years. Lack of physical activity, along with many other modifiable risk factors, such as hypertension, obesity, atherosclerosis, and diabetes, contributes through vascular dysfunction to the development of adverse cerebrovascular events in the future and has always been a topic of interest in the fields of neurology and stroke rehabilitation. We wrote this review article to elaborate on this relationship in detail. This article suggests that the physical activity role in stroke development and the rehabilitation process has a diverse role, where individuals with low physically active occupations are prone to develop a stroke more readily in comparison with other workers who have a moderate amount of physical activity in their jobs; however, less mobility appeared to be harmful too soon after stroke. In addition, we elucidate the effects of physical activity on sympathetic activity and remodeling of vascular response. Alterations in the neuroendocrine system include several factors. This includes harmful changes caused by increasing levels of epinephrine and norepinephrine. These changes are seen with stress-induced cerebrovascular injury and are often elevated in post-stroke patients. In contrast, post-stroke patients engaged in physical activity may prevent these harmful neurotrophic factors by reducing the elevated levels of epinephrine and norepinephrine. However, we need more studies in the near future to further explore this association process. Therefore, we recommend more research to explore the relationship of occupation-related factors and adverse stroke outcomes.

## Introduction and background

As physicians, we are most often asked two questions after a patient suffers from a stroke: “How am I going to return to my work, pay my bills, and get my life back in order?” and "What caused my stroke?” Many patients, especially young adults, are surprised to learn that their current job may actually have contributed to the risk factors leading to stroke, particularly since their occupation is often directly related to their lifestyle and is one of the determinants as to how much daily physical activity and inactivity is involved. Thus, the understanding that one's occupation may be a stroke-related risk is an issue that physicians often need to address with their patients in clinical practice. According to Harvey Cushing, “A physician is obligated to consider more than a diseased organ, more even than the whole man - he must view the man in his world." There is a rising concern to understand the risk factors of stroke in young adults due to the high negative impact on the productivity of young adults, mainly because it has a disproportionately large economic impact by leaving victims disabled during their most productive years [1]. Furthermore, while there are rising figures of stroke incidence in young adults (ages: 20 - 54) [2], studies have shown that etiology remains unknown in 30% - 40% of these patients. However, modifiable risk factors, such as physical activity, account for a large part of stroke occurrence in younger adults with four risk factors (high blood pressure, diabetes, smoking, and high LDL (low-density lipoprotein) levels) explaining almost 80% of stroke risk [3]. Regular physical activity can help lower elevated blood pressure, reduce LDL, and better regulate blood glucose in diabetes.

The first presentation of losing motor control over a body part or changing sensation in a young adult is very worrisome and would be considered an alarming sign to seek medical attention; the patient usually requests to know about the causes, as well as risk factors, of the condition. In fact, risk factors related to lifestyle and physical activity are becoming more popular among the general population. Moreover, there is a growing concern about the health aspects of the work type and working conditions.

There is a growing interest in identifying stroke risk factors linked to performing certain types of jobs, e.g., jobs with high physical demand such as construction and mining jobs. Furthermore, returning stroke patients to these jobs may intervene in the rehabilitation process. For instance, low physical activity jobs and a high-strain work environment could have a specific role initially, as well as an assessment of recovery post-stroke. This paper explores whether there is a significant role or not in this regard, as well as expected clinical implications of this role.

## Review

There has been increasing interest in the rising figures of stroke incidents in working young adults. According to one transversal retrospective study which analyzed electronic medical records (EMR) data during a four-year period for 134 patients aged 18 - 45, the average age of stroke patients appeared to be 33 years, and the majority were females (56%) [4]. However, the proportion of the first-ever stroke in young adults differed from country to country, ranging from < 5% to 20% of all strokes [1]. Another issue is multiple and variable risk factors leading to stroke in young adults which may require an individualized approach. One study examining the contribution of established potentially modifiable cardiovascular risk factors to the burden of stroke in young adults concluded that there were eight risk factors which explained 78.9% (95% confidence interval (CI), 76.3 - 81.4) of all strokes. However, low physical activity and hypertension were the most critical risk factors, accounting for 59.7% (95% CI, 56.3 - 63.2) and 27.1% (95% CI, 23.6 - 30.6), respectively, of all strokes [3].

Work environment has changed remarkably during the last two decades, and with more technological advancement in work fields, there is less need for physical effort at most types of jobs. As a result, less physically active job types are increasing. This, therefore, may contribute to obesity and thereby increase stroke risks. Furthermore, there is the additional challenge for workers in operations to maximize their work output. Therefore, an additional mental strain is added to perform well in current work fields. For instance, according to a meta-analysis study done using three databases (Pubmed, Embase, and PsychINFO), there were six prospective cohort studies comprising a total of 138,782 participants. The results showed an increase in stroke risk with high-strain jobs (relative risk (RR) 1.22, 95% CI 1.01 - 1.47) and were significant in women (RR 1.33, 95% CI 1.04 - 1.69) in comparison to men (RR 1.26, 95% CI 0.69 - 2.27). There was a lower risk with low-strain jobs regardless of gender type [5].

Physical activity/vascular accidents relationship model

There is a diverse relationship between physical activity and vascular accidents. For instance, one study about the relationship of physical activity and vascular incidents concluded that an inverse relationship was evident: the more the patient was physically active, the less he/she would develop vascular accidents, particularly peripheral vascular accidents [6]. The study analyzed the data of 16,446 patients using univariate and multivariate-adjusted logistic regression analysis. The data included the occupational physical activity intensity, measurement of the brachial-ankle index, health history, physical examinations, medication use, and blood biochemistry of the 16,446 subjects from nine areas throughout China. They classified the jobs into five categories depending on physical activity intensity of the job (jobless, very light, light, moderate, and heavy intensity). The results of the analysis showed that a steady reduction of peripheral vascular disease was correlated with an increase in physical activity intensity. When compared to the jobless group, the odds ratio (OR) for the other study groups was 0.65 (95% CI: 0.52, 0.82), 0.70 (95% CI: 0.56, 0.87), 0.57 (95% CI: 0.44, 0.73), and 0.65 (95% CI: 0.53, 0.80), respectively. However, when an analysis was done with an adjustment for gender, age, smoking, obesity/overweight, history of hypertension, hypercholesterolemia, diabetes, cardiovascular disease, and stroke, it showed OR 1.02 (95% CI: 0.80, 1.31), 0.91 (95% CI: 0.72, 1.15), 0.92 (95% CI: 0.70, 1.19), and 0.90 (95% CI: 0.72, 1.12), respectively (P trend < 0.05). The conclusion of this study attributed to occupational physical activity and peripheral vascular disease. However, direct analysis between occupational physical activity and stroke, specifically, was not included in this study [6]. 

The role of stress in vascular accidents

**Figure 1:**
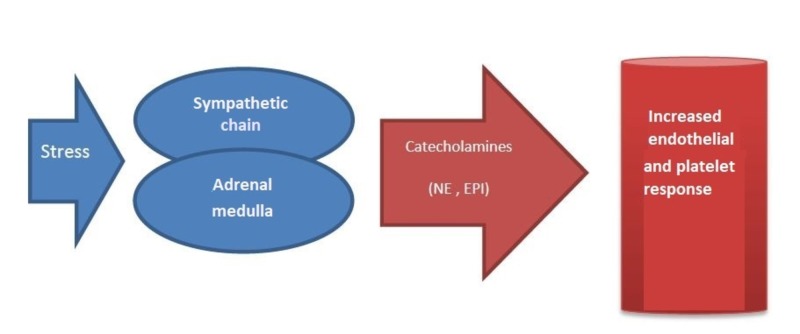
Process involving vascular accidents NE: norepinephrine; EPI: epinephrine

In their systemic review article, Huang et al. revealed their findings of the molecular contribution of acute stress hormones with vascular accidents, particularly cardiovascular accidents, in 2013, four years after a similar review. The article relied on sympathetic-adrenal-medullary (SAM) axis activation where the catecholamines, norepinephrine (NE) and epinephrine (EPI), played a significant role in cardiovascular response and rising vascular accident risks, which we elaborated in Figure 1 below [7].

In contrary, physical activity as a stress relieving factor also assessed in associated with a reduction of mortality according to a prospective cohort study that surveyed 13,241 person-months with a follow-up period of 71.96 months. Primarily, physical activity measured with accelerometry (ActiGraph 7164, ActiGraph, LLC, Pensacola, FL) and stroke incidents were reported with self-report of physician diagnosis. Interestingly, it showed for every 60 minutes/day increase in total physical activity there was a reduction of 28% of all-cause mortality related to stroke [8]. Additional validation for the positive impact of physical activity on stroke risk, as well as the degree of insult, was revealed in a subanalysis of data from a randomized clinical trial done in 2017 where patients with high pre-stroke physical activity (mainly treated with t-PA) had a reduction in the size of infarct growth in 24 hours, as well as the final infarct size [9].

Post-stroke occupational physical activity

The predictive factors for recovery potential were enhanced in relation to the motor function recovery process, and the algorithm utilized to support this finding was the Predict Recovery Potential (PREP) algorithm, where it modified therapy content and increased rehabilitation efficiency after stroke without compromising clinical outcome [10]. Therefore, prediction models for motor function recovery, particularly for the upper limb (UL), were revealed by a cross-sectional study that included 104 stroke patients with first-ever stroke and impaired motor function in the upper extremity. The findings were that models including grip strength or finger extension gave the most accurate predictions, with an overall predictive ability of 90.4% (95% CI 0.847 - 0.961) and a sensitivity of 92.9% (95% CI 0.851 - 1.0) and 90.5% (95% CI 0.816 - 0.979), respectively [11]. Recovery time and ability to regain motor function were used to guide rehabilitation and post-stroke recommendations. For instance, when the insult occurs in the brain white matter, we expect a poorer recovery over time [12]. However, some genetic variation and behavioral factors showed a positive influence on recovery (for instance, compensatory movement strategies developed after motor impairment through neural remodeling responses) [13]. When the condition became chronic, the similar use of dominant and nondominant ULs was evident, according to the Bailey et al. 2015 study [14].

The role of physical activity required in a job type not only influences stroke management and rehabilitation, it has, in addition, an important role in stroke prevention [15]. Furthermore, it augments public health efforts in the alleviation of long-term impact, especially among older workers [16]. Moreover, the nature of the job activity also helps in the management of the post-stroke state. For instance, during the recovery period from stroke, physical activity alone is considered an essential factor for improving motor functions, and this conclusion is confirmed through measurement of the brain-derived neurotrophic factor (BDNF). In fact, neuroplasticity recovery appeared to be positively influenced by aerobic exercise and also associated with the rise in BDNF [17].

The extreme form of physical activity (fast and strong action in a short duration) in some job types, e.g., mining jobs, has another positive influence in post-stroke skills retention. In fact, one study including 22 motor stroke patients showed that a single bout of high-intensity interval training performed for 15 minutes immediately after motor practice improved skill retention, thus, potentially accelerating motor recovery in these individuals [18].

Occupational choice in stroke

There was one study done to elucidate the relationship between exercise and brain tissue injury after cerebral ischemia in adult rats [19]. It showed that after clamping the middle cerebral artery of rats and subjecting them to exercises in a specific time frame there was a rising expression of pro-inflammatory cytokines (intercellular adhesion molecule-1 (ICAM-1), vascular adhesion molecule-1 (VCAM-1), tumour necrosis factor (TNF)-α, and interleukin (IL)-1β), as well as cell stress markers (heat shock protein 70 (HSP70) and hypoxia-inducible factor 1α (HIF-1α)), within three days post-clamping; however, there was less expression of these molecules if the patient began exercising after three days post-insult. While the physician's decision on returning patients to the same pre-stroke job is judged by the patient's ability to perform the job functions, the degree of physical activity allowed in the specific job should be timed so that it does not occur too early after the insult. 

Also, motor function and cognitive task ability required to achieve the tasks influence the choice of the job after stroke. Interestingly, according to a cross-sectional study done in 2017, patients with chronic stroke showed that cognitive tasks had a more significant influence than the motor tasks when patients were assessed during the completion of the study requirement. In other words, patients with chronic stroke tend to prioritize task accuracy and completion over maintaining activity speed [20].

## Conclusions

Physical activity has a significant role in the development of stroke in working adults. With the development of more technical tools, the demand for ambulation in the work field is becoming a hindrance for stroke prevention. Therefore, figures are rising in young adults across different age groups, especially females for a yet unknown reason. High-strain jobs requiring extreme physical activity may contribute in a harmful way, leading to vascular remodeling through neuroendocrine pathways, namely, through the process of epinephrine and norepinephrine. However, as seen during post-stroke recovery in patients undergoing physical activity, a reduced level of these harmful elevations of epinephrine and norepinephrine are seen along with improved motor function through neuroplasticity and elevated levels of BDNF. However, animal studies with rats suggest that the timing of return to a more physically active state is crucial in preventing further damage from pro-inflammatory cytokines (ICAM-1, VCAM-1, TNF-α, and IL-1β) and cell stress markers (HSP70 and HIF-1α). The findings are inconclusive because some studies are animal-based. Additional human-based studies are needed to strengthen the evidence quality. With all the evidence mentioned above, job nature recommendations for stroke patients and rehabilitation regarding the role of physical activity remains inconclusive. We will have to wait for additional data in the coming decades to understand more about this association.
